# A Rapid Self−Assembling Peptide Hydrogel for Delivery of TFF3 to Promote Gastric Mucosal Injury Repair

**DOI:** 10.3390/molecules29091944

**Published:** 2024-04-24

**Authors:** Jialei Chen, Jing Luo, Di Su, Na Lu, Jiawei Zhao, Zhongli Luo

**Affiliations:** 1 Molecular Medicine and Cancer Research Center, College of Basic Medical Sciences, Chongqing Medical University, Chongqing 400016, China; chenjialei0917@163.com (J.C.); sd199877@163.com (D.S.); lunar@stu.cqmu.edu.cn (N.L.); zjw980312@163.com (J.Z.); 2 Department of Pathology and Pathophysiology, Chongqing Medical University, Chongqing 400016, China; luojing668yeah@163.com

**Keywords:** self−assembly peptide, TFF3, gastric mucosal injury, gastric mucosal repair, drug delivery

## Abstract

Self-assembled peptide-based nanobiomaterials exhibit promising prospects for drug delivery applications owing to their commendable biocompatibility and biodegradability, facile tissue uptake and utilization, and minimal or negligible unexpected toxicity. TFF3 is an active peptide autonomously secreted by gastric mucosal cells, possessing multiple biological functions. It acts on the surface of the gastric mucosa, facilitating the repair process of gastric mucosal damage. However, when used as a drug, TFF3 faces significant challenges, including short retention time in the gastric mucosal cavity and deactivation due to degradation by stomach acid. In response to this challenge, we developed a self−assembled short peptide hydrogel, Rqdl10, designed as a delivery vehicle for TFF3. Our investigation encompasses an assessment of its properties, biocompatibility, controlled release of TFF3, and the mechanism underlying the promotion of gastric mucosal injury repair. Congo red/aniline blue staining revealed that Rqdl10 promptly self-assembled in PBS, forming hydrogels. Circular dichroism spectra indicated the presence of a stable β-sheet secondary structure in the Rqdl10 hydrogel. Cryo-scanning electron microscopy and atomic force microscopy observations demonstrated that the Rqdl10 formed vesicle-like structures in the PBS, which were interconnected to construct a three-dimensional nanostructure. Moreover, the Rqdl10 hydrogel exhibited outstanding biocompatibility and could sustainably and slowly release TFF3. The utilization of the Rqdl10 hydrogel as a carrier for TFF3 substantially augmented its proliferative and migratory capabilities, while concurrently bolstering its anti-inflammatory and anti-apoptotic attributes following gastric mucosal injury. Our findings underscore the immense potential of the self-assembled peptide hydrogel Rqdl10 for biomedical applications, promising significant contributions to healthcare science.

## 1. Introduction

Hydrogels, composed of hydrophilic polymers, possess a distinctive three-dimensional structure and retain substantial water content [1]. Over recent decades, molecular hydrogels have garnered significant interest as promising biomaterials with diverse applications in biomedicine and nanomedicine, including but not limited to cell culture [2], tissue engineering [3], cancer therapy [4], drug delivery [5], and regenerative medicine [6]. Peptides consist of short sequences of amino acids, and their self-assembly refers to the process whereby peptide molecules spontaneously form specific nanostructures [7]. The amino acid residues carried by self-assembling peptides can drive the self-assembly of peptides into stable, low-energy structures through non−covalent interactions, including hydrogen bonding, electrostatic forces, hydrophobic/hydrophilic interactions, π-π stacking, and van der Waals forces. Peptide molecules, when rationally designed, can self-assemble into specific nanostructures with rich hierarchical levels under varying conditions such as pH, temperature, and ionic strength, including vesicles [8], nanotubes [9], nanofibers [10], and hydrogels [7]. Peptide-based nanogel systems, owing to their exceptional biocompatibility and biodegradability, have been widely utilized as ideal delivery vehicles for various drugs [11]. Self-assembling peptide hydrogel carriers can effectively address issues such as low water solubility, instability, uneven biological distribution, high degradation rates, and lack of targeted functionality of drugs, thereby enhancing the bioavailability and therapeutic efficacy of the medications [12]. Currently, self-assembling peptide hydrogel delivery systems have successfully achieved precise delivery and controlled release of a variety of peptide-based active pharmaceuticals. For instance, the RAD16-II self-assembling peptide nanogel has been employed for the intramyocardial delivery of platelet-derived growth factor (PDGF) and vascular endothelial growth factor (VEGF), aimed at promoting revascularization in the myocardial infarction areas of rats and pigs, while effectively avoiding issues such as pulmonary hypertension caused by the systemic administration of PDGF [13,14]. Similarly, employing RADA16 self-assembling peptide hydrogel to encapsulate recombinant Bone Morphogenetic Protein-2 (BMP-2) not only effectively stores and releases BMP-2 but also preserves its biological activity, significantly enhancing the stability of BMP-2. This strategy has been successfully applied to the healing of bone defects [15].

Gastric mucosal injury is common in daily life [16]. Typically, the damaged gastric mucosa does not always heal quickly within a short period, and the damage may even be accompanied by potential serious complications such as bleeding or perforation [17]. Due to its diverse etiology, achieving the treatment and complete cure of gastric mucosal injury poses significant challenges. Currently, the primary approaches to promoting the repair of gastric mucosal injury involve inhibiting gastric acid secretion, administering anti-Helicobacter pylori therapy, and utilizing gastric mucosal protectants [18]. However, these medications may induce various side effects, including drug resistance, allergic reactions, and abnormal heart rhythms [19]. Hence, identifying a natural active ingredient with high safety, minimal side effects, and enhanced efficacy is imperative for promoting the repair of gastric mucosal injury.

The maintenance of gastric mucosa integrity is reliant on the concerted action of numerous secreted factors, among which the secretion of trefoil factor 3 (TFF3) plays a crucial role [20]. TFF3 is a small peptide rich in cysteine, comprising 59 amino acids. Upon maturation, TFF3 is secreted and folded into a characteristic cloverleaf-like compact tricyclic structure, formed by six conserved cysteine residues linked by three disulfide bonds [21]. While TFF3 expression is typically challenging to detect in normal gastric mucosa, the robust expression of TFF3 can be observed in the periulcer tissues of gastric ulcer patients and rats [22,23]. Animal studies have demonstrated that TFF3 administration mitigates gastric mucosal injury induced by various factors including alcohol [24], NSAIDs [25], stress [26], and lipopolysaccharide [27]. The potential roles of TFF3 encompass the maintenance of gastric pH, stimulation of gastric mucosal healing, and enhancement of gastric mucosal defense [25,28]. Notably, unlike many gastroprotective agents, the gastric mucosal protective effect of TFF3 operates independently of prostaglandins and is realized at the surface of the gastric mucosal lumen [28]. Different routes of administration can markedly influence the efficacy of TFF3 in disease treatment. In a rat model of colitis, the intestinal administration of TFF3 has demonstrated greater efficacy compared to systemic administration. Conversely, the subcutaneous injection of TFF3 monomer has been shown to exacerbate colitis induced by mitomycin C or dextran sulfate sodium salt [29]. Previous investigations have highlighted the characteristic cloverleaf structure of TFF3, which imparts stability against digestion by protein hydrolases, thereby facilitating its role in the luminal environment of the digestive tract [20,21,30,31]. However, recent findings indicate that TFF3, along with its homodimeric and cloverleaf domains, are susceptible to degradation and inactivation in an acidic environment containing pepsin [32,33,34]. In response to this formidable challenge, Nayara and collaborators utilized chemical synthesis methodologies to generate TFF3 analogs, notably TFF3 (C57Acm), TFF310-50, and TFF37-54, exhibiting TFF3 activity and distinctive trilobal architectures tailored to fortify stability in the gastrointestinal tract [35]. Nevertheless, drug modification encounters multifarious obstacles, including subtle structural alterations capable of eliciting profound discrepancies in drug efficacy, pharmacokinetics, and toxicity profiles. Another auspicious strategy involves the development of apt drug delivery systems aimed at augmenting stability, solubility, and targeting precision, thereby amplifying therapeutic efficacy while curtailing adverse effects [36].

TFF3 is an active peptide with various biological functions, acting on the gastric mucosal surface to facilitate the repair process of gastric mucosal damage. However, when used as a drug, TFF3 faces significant challenges, including short retention time in the gastric mucosal cavity and deactivation due to degradation by stomach acid. To address these issues, our research team innovatively synthesized a new chiral self-assembling peptide hydrogel, Rqdl10, for the delivery of TFF3. Furthermore, the involvement and underlying mechanism of TFF3@Rqdl10 in the repair of gastric mucosal injury were elucidated through a combination of in vivo and in vitro experiments. Our findings offer novel perspectives and insights into advancing the repair of gastric mucosal injury.

## 2. Results

### 2.1. Characteristics of Rqdl10 Hydrogel

**Figure 1:**
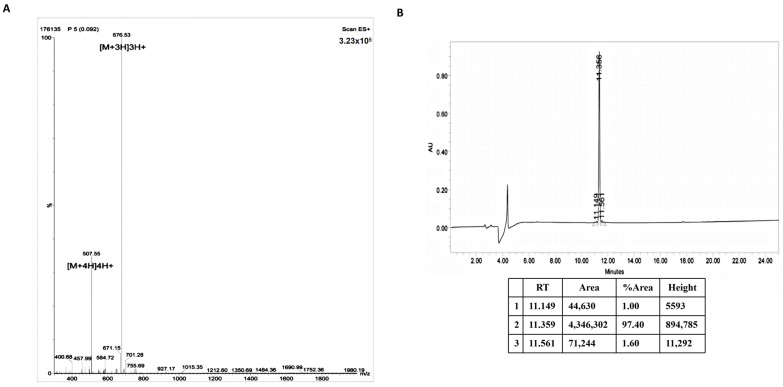
Mass spectrometry and HPLC analysis of peptide Rqdl10. (**A**) Mass spectrometry analysis of peptide Rqdl10; (**B**) HPLC analysis of peptide Rqdl10.

**Figure 2:**
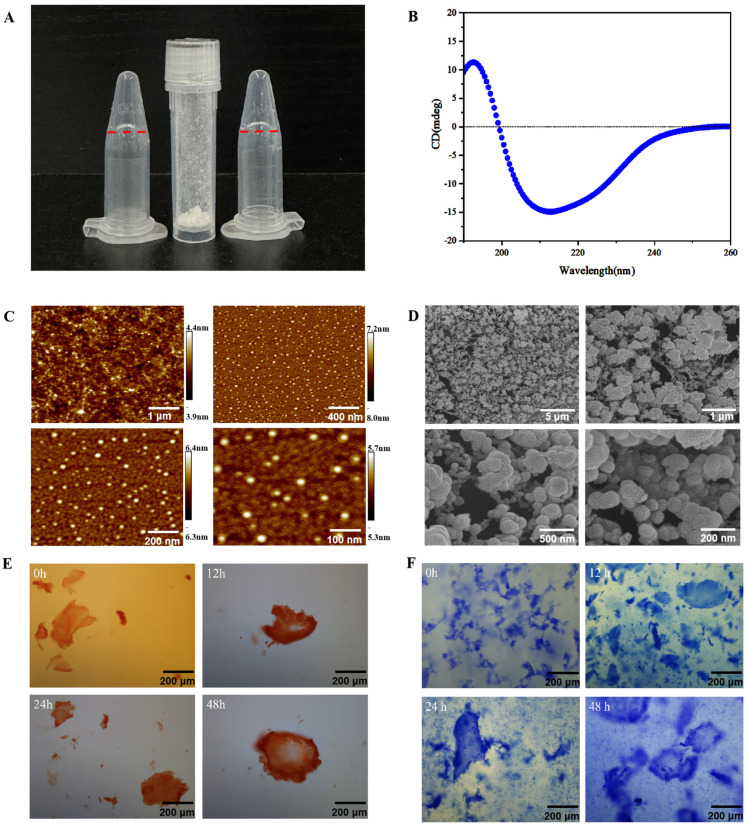
Characterization of Rqdl10 hydrogel. (**A**) Hydrogel formed by self−assembling peptide Rqdl10,The red line represents the lower edge of Rqdl10 hydrogel; (**B**) Circular dichroic analysis of Rqdl10 hydrogel; (**C**) Atomic force microscopy (AFM) analysis of Rqdl10 hydrogel; (**D**) Cryogenic scanning electron microscopy (cryo-SEM) analysis of Rqdl10 hydrogel; (**E**) Congo red staining results of Rqdl10 hydrogel at 0 h, 12 h, 24 h and 48 h; (**F**) aniline blue staining results of Rqdl10 hydrogel at 0 h, 12 h, 24 h and 48 h.

The amino acid sequence of Rqdl10, RQDLKTEIRYDFKADE-NH2, is a linear peptide from the N-terminus to the C-terminus. The relative molecular mass of Rqdl10 was determined to be 2026.2 (Figure 1A), with a purity of 97.40% (Figure 1B). The Rqdl10 solution can self-assemble into a hydrogel upon ion triggering through hydrogen bonding, van der Waals forces, aromatic stacking, charge–charge interactions, and hydrophobic–hydrophilic interactions [7] (Figure 2A). Circular dichroism spectroscopy revealed that the Rqdl10, after 24 h of self-assembly, exhibited a positive peak at 192.5 nm and a negative peak at 211.5 nm, indicative of the formation of a typical β-sheet structure (Figure 2B). Atomic force microscopy (AFM) analysis demonstrated the formation of vesicle-like structures by the Rqdl10 after 24 h of self-assembly, with diameters ranging from 100 to 150 nm and an average diameter of 127 nm. The vesicles were interlaced with each other to form vesicle-like three-dimensional nanostructures (Figure 2C). The cryo-scanning electron microscopy results revealed that the Rqdl10 hydrogel exhibited a vesicular morphology whose nanostructures connected to each other in the field of view of 200 nm and 500 nm. Upon expanding the field of view to 1 μm and 5 μm, it was observed that the vesicular nanostructures intertwined to form a three-dimensional network structure (Figure 2D). Additionally, the Congo red/aniline blue staining of the Rqdl10 hydrogel indicated the presence of an evacuation film structure at 0 h of self-assembly. However, with an extension of self-assembly time to 12, 24, and 48 h, this structure transformed into a denser, lamellar, and more stable configuration (Figure 2E,F).

### 2.2. Rqdl10 Hydrogel Delivery of TFF3

**Figure 3:**
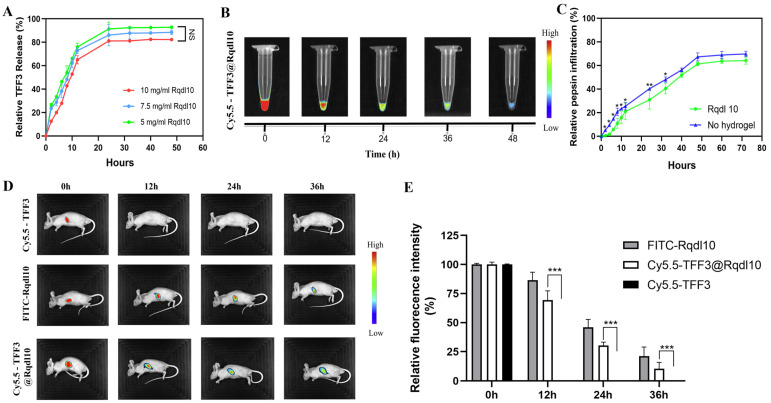
Rqdl10 hydrogel delivery of TFF3. (**A**) In vitro release curve of TFF3 from different concentrations of Rqdl10 hydrogel in PBS; (**B**) In vitro imaging of TFF3 in Rqdl10 hydrogel; (**C**) Sequestration effect of Rqdl10 hydrogel on pepsin. * *p* < 0.05, ** *p* < 0.01,Comparison between Rqdl10 hydrogel group and no hydrogel group; (**D**) Representative images of optical in vivo imaging; (**E**) Quantification analysis of the fluorescence intensity retention in stomach. *** *p* < 0.001. Statistics for each group are expressed as mean ± SD (*n* = 4).

To assess the controlled release efficacy of the Rqdl10 hydrogel, we investigated its effect on the release of TFF3. Our findings indicate that while there were slight variations in the controlled release of TFF3 among different concentrations of the Rqdl10 hydrogel, the TFF3@Rqdl10 solution achieved a near-complete release of TFF3 after 24 h. Specifically, the controlled release of TFF3 by the Rqdl10 hydrogel was determined to be 92.3%, 87.2%, and 81.1% at concentrations of 5 mg/mL, 7.5 mg/mL, and 10 mg/mL, respectively (Figure 3A). Given the optimal stability observed with 10 mg/mL Rqdl10 hydrogel for the controlled release of TFF3, we selected this concentration as the delivery vehicle for TFF3. Similarly, in vitro imaging of TFF3 within the Rqdl10 hydrogel demonstrated that TFF3 could be retained in Rqdl10 hydrogels for more than 36 h (Figure 3B). Given that the concentration of pepsin in the stomach fluctuates between 0.5 and 1.0 mg/mL, we utilized a 2 mg/mL pepsin solution (dissolved in pH = 3 buffer) to simulate the intragastric environment. Our results revealed that the Rqdl10 hydrogel significantly attenuated the diffusion of pepsin from the upper chamber to the lower chamber within 32 h compared to the no hydrogel group (Figure 3C). In the in vivo experiments, both FITC−Rqdl10 and Cy5.5−TFF3 in Cy5.5−TFF3@Rqdl10 can be detected and kept in the stomach from 0 h to 36 h after injection, while no detectable signal of Cy5.5−TFF3 was observed at 12 h after injection (Figure 3D,E). This indicates that TFF3@Rqdl10 can effectively adhere to the surface of the gastric mucosa, efficiently sequestering pepsin while sustainably and slowly releasing TFF3.

### 2.3. Biocompatibility of Rqdl10

**Figure 4:**
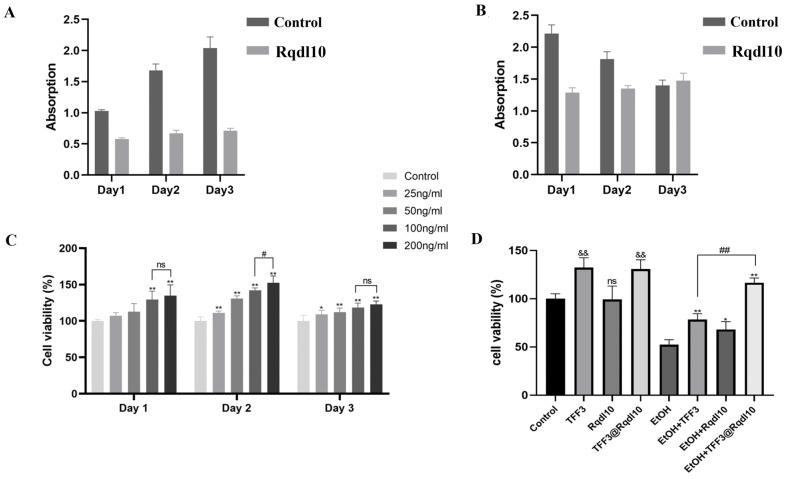
Determination of cell viability of GES−1 under different treatments. (**A**) Proliferation assay of GES-1 cells in Rqdl10 hydrogel matrix; (**B**) Cytotoxicity assay of GES-1 cells in Rqdl10 hydrogel matrix; (**C**) Optimal concentration of TFF3 to promote GES-1 proliferation,* *p* < 0.05,** *p* < 0.01 (vs. control group), # *p* < 0.05, ns means no statistical significance; (**D**) Effect of TFF3, Rqdl10 hydrogel, and TFF3@Rqdl10 treatment on GES-1 cell viability in the presence or absence of ethanol. && *p* < 0.01 (vs. control group), * *p* < 0.05, ** *p* < 0.01 (vs. EtOH Group), ## *p* < 0.01. Statistics for each group are expressed as mean ± SD (*n* = 6).

The assessment of biocompatibility is pivotal in determining the suitability of hydrogels for biomedical applications. To ascertain the biocompatibility of the Rqdl10 hydrogel, the cell viability of GES1 cells was evaluated using a cell proliferation toxicity assay employing the CCK8 method. The results of the cell proliferation assay revealed that the GES1 cells in the control group exhibited continuous growth over the initial 3 days. In contrast, the proliferation of GES1 cells within the Rqdl10 hydrogel matrix was slower; however, they remained in a state of slow proliferation, with no discernible decline in cell activity observed (Figure 4A). Furthermore, cytotoxicity assessments demonstrated that the GES1 cells in the control group exhibited the highest cell activity on day one, followed by a gradual decrease thereafter. Conversely, the activity of GES1 cells within the Rqdl10 hydrogel matrix remained relatively stable, displaying a slight increasing trend (Figure 4B). These findings collectively suggest that the Rqdl10 hydrogel exhibits favorable biocompatibility.

### 2.4. TFF3@Rqdl10 Promoted Cell Proliferation and Migration

**Figure 5:**
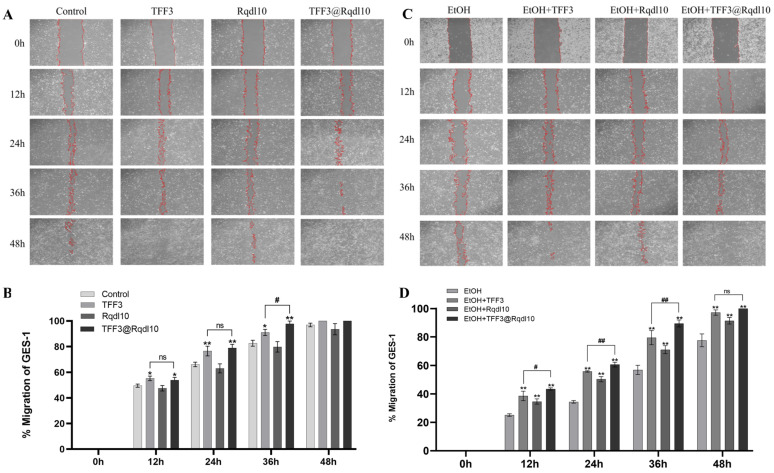
Effect of TFF3, Rqdl10 hydrogel, and TFF3@Rqdl10 treatment on GES−1 cell migration in the presence or absence of ethanol. (**A**) The scratch assay on GES−1 cell migration in treatment with TFF3, Rqdl10 hydrogel, and TFF3@Rqdl10 at different incubation times; (**B**) Migration rate of GES-1 cells after treatment with TFF3, Rqdl10 hydrogel, and TFF3@Rqdl10, * *p* < 0.05, ** *p* < 0.01 (vs. control group), # *p* < 0.05, ns means no statistical significance; (**C**) The scratch assay on GES−1 cell migration in treatment with TFF3, Rqdl10 hydrogel, and TFF3@Rqdl10 at different incubation times in ethanol environment; (**D**) Migration rate of GES−1 cells after treatment with TFF3, Rqdl10 hydrogel, and TFF3@Rqdl10 in ethanol environment.** *p* < 0.01 (vs. EtOH Group), # *p* < 0.05, ## *p* < 0.01, ns means no statistical significance. Statistics for each group are expressed as mean ± SD (*n* = 4).

The GES-1 cells were exposed to various concentration gradients of TFF3 for 24 h to determine the optimal environment. Cell viability was assessed using the CCK8 assay after 24 h of treatment. The results revealed that a concentration of 200 ng/mL of TFF3 was optimal for promoting the proliferation of GES-1 cells (Figure 4C). Based on these findings, we selected a concentration of 200 ng/mL of TFF3 for subsequent experiments. In an ethanol-free environment, treatment with TFF3 and TFF3@Rqdl10 significantly enhanced GES-1 cell proliferation, whereas treatment with Rqdl10 hydrogel alone showed no such effect. Conversely, in the presence of ethanol, the viability of the GES-1 cells in the EtOH group decreased to 52.45% of the control group. However, pre-treatment with TFF3, Rqdl10 hydrogel, and TFF3@Rqdl10 increased cell viability to 78.57%, 68.17%, and 116.71%, respectively (Figure 4D). In Figure 5A, both the TFF3 and TFF3@Rqdl10 treatments demonstrated significant enhancement in promoting GES-1 cell migration, whereas the Rqdl10 hydrogel treatment did not exhibit a similar effect (Figure 5B). Furthermore, we assessed cell migration under ethanol-stimulated conditions. The results revealed that following treatment with TFF3, Rqdl10 hydrogel, and TFF3@Rqdl10, the rate of the scratch closure of the GES-1 cells was notably faster compared to the EtOH group within 48 h (Figure 5C). Notably, the TFF3@Rqdl10 treatment significantly facilitated GES-1 migration after ethanol-induced damage more effectively than TFF3 alone (*p* < 0.01) (Figure 5D). These findings suggest that while the Rqdl10 hydrogel treatment mitigated ethanol-induced injury to the GES-1 cells, it did not promote their proliferation and migration. Conversely, the TFF3 treatment promoted both the proliferation and migration of the GES-1 cells, while the TFF3@Rqdl10 treatment not only reduced the ethanol-induced injury to the GES-1 cells but also enhanced their proliferation and migration.

### 2.5. In Vitro Anti-Apoptosis and Anti-Inflammatory Activity of TFF3@Rqdl10

**Figure 6:**
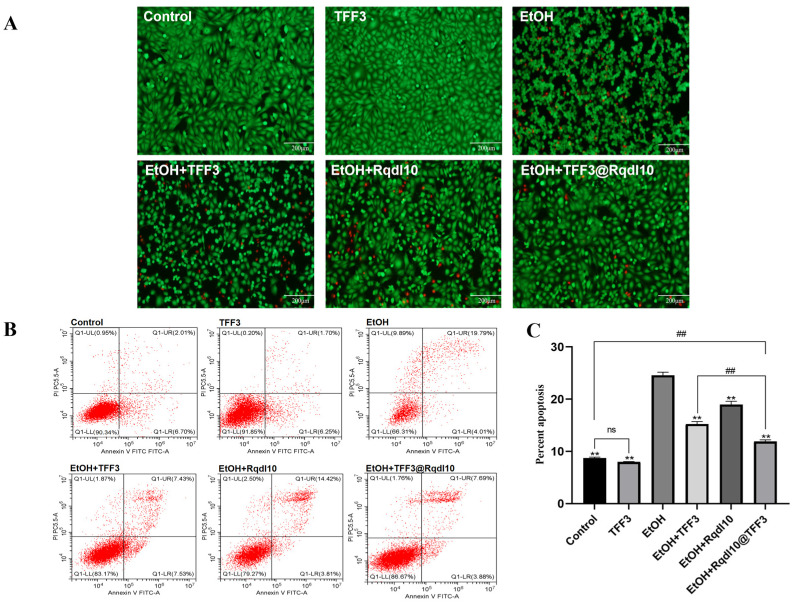
Apoptosis detection in GES-1 cells. (**A**) The live/dead staining of GES-1 cell treatment with TFF3, Rqdl10 hydrogel, and TFF3@Rqdl10; (**B**) Flow cytometric analysis for quantification of apoptotic cells; (**C**) Percentage of apoptotic GES−1 cells. ** *p* < 0.01 (vs. EtOH Group); ## *p* < 0.01; ns means no statistical significance. Statistics for each group are expressed as mean ± SD (*n* = 4).

**Figure 7:**
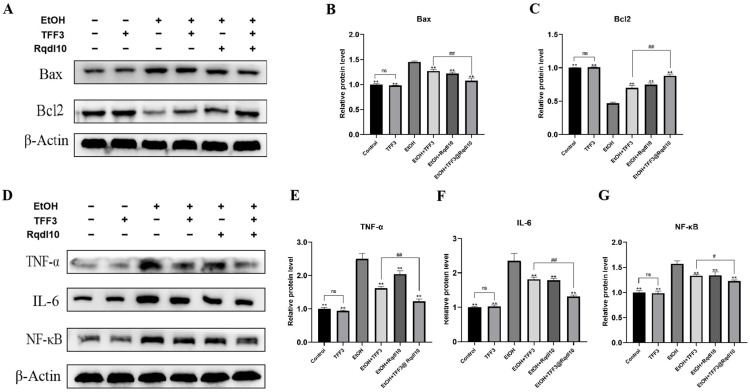
The impact of TFF3@Rqdl10 on apoptosis and inflammation-related protein expression in GES−1 cells after ethanol-induced injury. (**A**) WB analysis of Bax and Bcl2 in GES-1 cells after ethanol injury. Relative protein level of Bax (**B**) and Bcl2 (**C**), ** *p* < 0.01 (vs. EtOH group), ## *p* < 0.01, ns means no statistical significance; (**D**) WB analysis of TNF-α, IL-6, and NF-κB in GES-1 cells after ethanol injury; Relative protein level of TNF-α (**E**), IL-6 (**F**), and NF-κB (**G**), ** *p* < 0.01 (vs. EtOH group), # *p* < 0.05, ## *p* < 0.01, ns means no statistical significance. Statistics for each group are expressed as mean ± SD (*n* = 6).

Calcein-AM stains live cells green, while PI stains dead cells red. As shown in Figure 6A, under ethanol-free conditions, GES-1 cells exhibit a spindle-shaped and flattened morphology with no overlap or aggregation between cells. In the TFF3 group, the cell density increases compared to the control group. After ethanol treatment, the GES-1 cells change from spindle-shaped to spherical and aggregate, with numerous red-stained dead cells scattered throughout. In the EtOH + TFF3 and EtOH + Rqdl10 groups, a small number of GES-1 cells with normal morphology and a large number with irregular morphology are observed, with a slightly higher density of green-stained live cells than the EtOH group, and fewer red-stained dead cells than the EtOH group. The EtOH+TFF3@Rqdl10 group demonstrates the most potent anti-apoptotic ability, with the GES-1 cells exhibiting a predominantly normal morphology and only scattered red-stained dead cells. Flow cytometry was employed to assess the apoptosis of the GES-1 cells following ethanol injury (Figure 6B). Under normal conditions, the number of apoptotic GES-1 cells is minimal. The ethanol treatment significantly increased the apoptosis rate, reaching 24.58% for the GES-1 cells in the EtOH group. However, after pretreatment with TFF3, Rqdl10 hydrogel, and TFF3@Rqdl10, the apoptosis rate of the GES-1 cells decreased to 15.23%, 18.96%, and 11.9%, respectively (Figure 6C). Notably, the TFF3@Rqdl10 treatment exhibited a significant inhibitory effect on apoptosis, whereas the effect of the Rqdl10 hydrogel treatment was primarily observed in delaying the onset of apoptosis. Furthermore, Western Blotting results (Figure 7A–C) demonstrated that TFF3 did not alter the expression of Bax and Bcl2 in normal GES-1 cells (*p* > 0.05). In the ethanol injury model, ethanol induces the upregulation of Bax and downregulation of Bcl2 expression in the GES-1 cells. The expression levels of Bax and Bcl2 in the GES-1 cells treated with TFF3, Rqdl10 hydrogel, and TFF3@Rqdl10 significantly differed from those in the EtOH group (*p* < 0.01), indicating that all the treatments effectively inhibit the apoptosis of GES-1 cells. Additionally, there was a significant difference in the ability of the TFF3@Rqdl10 treatment to downregulate Bax and upregulate Bcl2 compared to TFF3 alone (*p* < 0.01). Next, we will shift our focus to the expression of intracellular inflammation-related proteins. Inflammation is an inevitable response when cells or tissues are injured. TNF-α and IL-6, as biomarkers of acute inflammatory response, are actively involved in several inflammatory processes and are closely associated with the severity of gastric mucosal injury [37]. On the other hand, NF-κB, as a major regulator of inflammatory response, plays an important role in promoting the transcription of TNF-α and IL-6. Therefore, we selected TNF-α, IL-6 and NF-κB to elucidate the mechanisms associated with the alleviation by TFF3@Rqdl10 of ethanol-induced acute gastric mucosal injury. The ethanol treatment resulted in the increased expression of pro-inflammatory factors such as TNF-α, IL-6, and NF-κB in the GES-1 cells (Figure 7D–G). However, the inflammatory state of the GES-1 cells was significantly ameliorated following the treatment with TFF3, Rqdl10 hydrogel, and TFF3@Rqdl10 compared to the ethanol group. Moreover, the anti-inflammatory and anti-apoptotic capacities of the GES-1 cells after the TFF3 treatment were found to be significantly weaker than those of the TFF3@Rqdl10 treatment (*p* < 0.01).

### 2.6. In Vivo Anti-Apoptosis and Anti-Inflammatory Activity of TFF3@Rqdl10 

**Figure 8:**
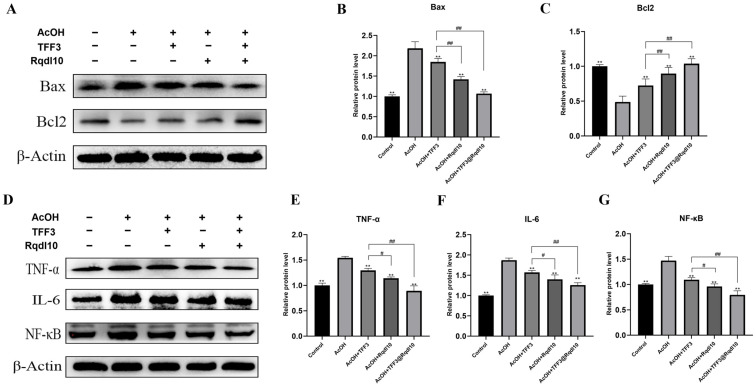
The impact of TFF3@Rqdl10 on apoptosis and inflammation−related protein expression after acetic acid injury in rat gastric mucosa. (**A**) WB analysis of Bax and Bcl2 in gastric tissue after acetic acid injury. Relative protein level of Bax (**B**) and Bcl2 (**C**), ** *p* < 0.01 (vs. EtOH group), ## *p* < 0.01, ns means no statistical significance; (**D**) WB analysis of TNF-α, IL-6, and NF-κB in gastric tissue after acetic acid injury; Relative protein level of TNF-α (**E**), IL-6 (**F**), and NF-κB (**G**), ** *p* < 0.01 (vs. AcOH group), # *p* < 0.05, ## *p* < 0.01, ns means no statistical significance. Statistics for each group are expressed as mean ± SD (*n* = 6).

TFF3, an active peptide secreted by gastric mucosal cells, possesses a variety of biological functions, and its action on the surface of the gastric mucosa helps to promote the repair process of gastric mucosal damage. However, when used as a drug, TFF3 faces significant challenges, including short retention time in the gastric mucosal cavity and deactivation due to degradation by stomach acid. To further investigate, we explored the effects of TFF3, Rqdl10 hydrogel, and TFF3@Rqdl10 treatment on the expression of inflammation and apoptosis-related proteins by establishing a rat acetic acid gastric mucosal injury model. As depicted in the Western Blotting results (Figure 8A–G), the TFF3, Rqdl10 hydrogel, and TFF3@Rqdl10 treatments all effectively suppressed the expression of TNF-α, IL-6, and NF-κB, while also inhibiting Bax expression and promoting Bcl2 expression. Interestingly, the Rqdl10 hydrogel treatment exhibited a significantly stronger inhibition of inflammatory and apoptotic responses compared to the TFF3 treatment in the in vivo experiments (*p* < 0.01). This difference may be due to the fact that TFF3 is easily lost and susceptible to degradation by stomach acid when locally applied to the site of gastric mucosal injury. In contrast, the Rqdl10 hydrogel can adhere to the surface of damaged gastric mucosa for an extended duration, reducing the continuous stimulation of gastric acid on the damaged mucosa and creating a favorable environment for gastric mucosal healing. In addition, treatment with TFF3@Rqdl10 demonstrated superior suppression of inflammatory and apoptotic responses compared to treatment with TFF3 alone. This implies that TFF3@Rqdl10 not only shields the damaged gastric mucosa from continuous gastric acid stimulation but also gradually releases TFF3 to facilitate the repair of gastric mucosal injury. These findings suggest that the Rqdl10 hydrogel can serve as an optimal vehicle for TFF3, thereby promoting the mending of gastric mucosal injury.

## 3. Discussion

Numerous drugs suffer from limited bioavailability and inadequate therapeutic effectiveness, primarily attributed to factors such as low water solubility, instability, suboptimal biodistribution, rapid degradation, and a lack of targeting specificity [38]. Presently, conventional drug delivery platforms encompass viruses, liposomes, polymers, and synthetic inorganic materials [39,40,41,42]. Self-assembled peptides emerge as promising candidates for drug delivery vehicles owing to their remarkable biocompatibility, biodegradability, facile tissue absorption and utilization, and minimal or absent unexpected toxicities [11]. Self-assembled short peptides utilize various molecular forces including hydrogen bonding, electrostatic interactions, hydrophilic–hydrophobic interactions, π-π interactions, and van der Waals forces generated by self−contained amino acid residues to encapsulate or adsorb drugs, thereby forming a stable drug delivery system [43]. These self-assembled peptide vehicles can enhance drug pharmacokinetics and therapeutic efficacy by facilitating the controlled release and precise delivery of drugs [44]. Additionally, they can improve drug bioavailability by responding to environmental cues for drug release and minimizing drug degradation during circulation [45]. Currently, the self−assembled short peptide delivery system has achieved the precise delivery and controlled release of various substances including hydrophobic drugs, peptides, nucleic acids, vaccines, and more [46].

The repair of gastric mucosal injury constitutes a complex and coordinated biological process, encompassing multiple critical steps to ensure the effective repair and functional restoration of damaged tissue [47]. Throughout this process, the proliferation and migration of injured mucosal cells, along with apoptosis and inflammatory responses, play pivotal roles. Proliferation and migration facilitate the formation of new cells, filling the damaged areas and establishing a complete cellular architecture, thus accelerating the healing of the injury site [48]. Following gastric mucosal damage, there is a rapid increase in cell apoptosis, leading to further tissue destruction [49]. By inhibiting apoptosis, excessive cell death can be prevented, aiding in the maintenance of tissue integrity and providing favorable conditions for repair [50]. The inflammatory response that follows closely typically dictates the outcomes of healing [51]. Inflammation subsequent to gastric mucosal injury unfolds in distinct phases to facilitate the restoration of tissue architecture. Initially, an early pro-inflammatory response orchestrates the recruitment of key inflammatory cells to initiate the repair cascade. Subsequently, this pro-inflammatory cascade begins to subside, concomitant with a phenotypic transition of macrophages to a reparative state. Finally, as inflammatory cells vacate the injury site or undergo apoptosis, tissue homeostasis is restored [52,53]. The microenvironment of chronic wounds is typically characterized by the accumulation of many pro-inflammatory macrophages, along with the upregulation of inflammatory mediators such as NF-κB, TNF-α, and IL-6 [54]. Chronic inflammation can lead to sustained tissue damage and even the formation of ulcers. By inhibiting the inflammatory response, the release of inflammatory mediators can be reduced, decelerating the process of tissue destruction and facilitating the creation of a conducive environment for repair [54]. Precisely regulating these responses and their interactions during the gastric mucosal injury repair process is an effective therapeutic strategy for enhancing the healing of gastric mucosal damage. TFF3 is an active peptide with multiple biological functions, playing roles not only in promoting the proliferation and migration of GES-1 cells but also exhibiting significant anti-inflammatory and anti-apoptotic properties. As a chiral self-assembling peptide hydrogel, Rqdl10 hydrogel has not yet been observed as having a definitive biological function. However, the Rqdl10 can self−assemble into a hydrogel, and through a physical barrier function similarly to gastric mucus, it effectively protects the gastric mucosa and diminishes the damage induced by ethanol and acetic acid. Our research also discovered that the therapeutic effect of the Rqdl10 hydrogel on the repair of gastric mucosal damage in in vivo models surpasses that of TFF3. The in vitro models reveal that TFF3’s protective effect against ethanol-induced gastric mucosal injury is superior to that of the Rqdl10 hydrogel. This phenomenon might be related to the fact that TFF3, when co-cultured with GES-1 cells under in vitro conditions, has its bioactivity and duration of action fully ensured. In contrast, when TFF3 is directly applied as a drug to the gastric mucosal cavity, its retention time and bioactivity in the stomach may not be guaranteed to the same extent. TFF3@Rqdl10 adheres to the gastric mucosa and prolongs the retention time of TFF3 in the stomach by slowly releasing TFF3. It also enhances the stability of TFF3 in the gastric mucosal cavity through the sequestration of pepsin. Compared to the use of TFF3 or Rqdl10 hydrogel alone, TFF3@Rqdl10 significantly enhances the reparative effects on gastric mucosal damage, more effectively facilitating the proliferation and migration of gastric mucosal cells, and inhibiting inflammation and apoptotic responses, thus offering multi-layered support for the repair of gastric mucosal injuries. These results indicate that TFF3@Rqdl10 is an effective strategy for treating gastric mucosal injuries.

## 4. Materials and Methods

### 4.1. Synthesis of Rqdl10

Rqdl10 was designed by our research team and synthesized by Chengdu Sciobio Surgery Institute (Chengdu, China). The amino acid sequence of Rqdl10 is RQDLKTEIRYDFKADE-NH2, constituting a linear polypeptide from the N-terminus to the C-terminus. The peptide’s molecular weight and purity were determined using mass spectrometry (LC3000, SHIMADZU, Kyoto, Japan) and HPLC (Nexera UHPLC, SHIMADZU, Kyoto, Japan), respectively. 

### 4.2. Hydrogel Preparation

A peptide solution was prepared by dissolving 20 mg of Rqdl10 in 1 mL of sterile water. Subsequently, 1 mL of PBS was added to the peptide solution to induce self-assembly of the hydrogel at room temperature for 24 h, denoted as Rqdl10 hydrogel. Rqdl10 was dissolved in a sterile aqueous solution of TFF3 and then added to PBS to self-assemble into a drug-loaded hydrogel at room temperature, denoted as TFF3@Rqdl10.

### 4.3. Characterization of Rqdl10 Hydrogel

To investigate the secondary structure of the Rqdl10 hydrogel, a JASCO J-815 Spectrometer (JASCO, Kyoto, Japan) was utilized for detection. AFM (multimode8, Bruker, Massachusetts, USA) was employed to acquire the surface topography and structural information for the Rqdl10 hydrogel. The three-dimensional structure of the Rqdl10 hydrogel was captured using cryo-SEM (SU8010, Hitachi, Kyoto, Japan). The gel formation was observed through Congo red/aniline blue staining at 0 h, 12 h, 24 h, and 48 h following Rqdl10 self-assembly.

### 4.4. Controlled Release of TFF3 by Rqdl10 Hydrogel In Vitro

TFF3@Rqdl10 was prepared following previously described methods. Briefly, 100 µL of TFF3@Rqdl10 (1 ug/mL TFF3) was dispensed into the bottom of a 96-well plate, followed by the addition of 100 µL of PBS to each well. The plate was then incubated at 37 °C and continuously agitated on a shaker. To ensure consistent initial conditions, all the drug-containing wells for various time points were prepared simultaneously. At specified time intervals (0 h, 2 h, 4 h, 6 h, 8 h, 10 h, 12 h, 24 h, 32 h, 40 h, 48 h), supernatants were collected from selected wells (*n* = 3), while others remained undisturbed. TFF3 concentrations were quantified using a commercial ELISA kit (Cusabio, Wuhan, China) as per the manufacturer’s protocol, and the release profile of Rqdl10 for TFF3 was determined.

### 4.5. Analysis of Pepsin Sequestration by Rqdl10 Hydrogel In Vitro

We prepared a 24-well plate in advance by adding 1100 µL of buffer (pH = 3.0). Then, we took 100 µL of the Rqdl10 hydrogel (10 mg/mL) and placed it at the bottom of the upper chamber of the transwell, gently shaking to ensure it lay flat. Next, we added 200 µL of pepsin solution (2 mg/mL, pH = 3.0) to the upper chamber and placed the smaller chamber in the buffer. We collected and replenished the buffer at 0 h, 2 h, 4 h, 6 h, 8 h, 10 h, 12 h, 24 h, 36 h, 48 h, 60 h, and 72 h time points. We measured the absorbance at 562 nm using the CBA method and calculated the percentage of pepsin that had infiltrated into the lower chamber using a standard curve.

### 4.6. In Vitro Imaging of Cy5.5−TFF3 in Rqdl10 Hydrogel

Cy5.5 dye was dissolved in DMF at a concentration of 12.56 mM. TFF3 (5 µg/µL) solution was prepared in pure water; then, Cy5.5 dye was added to the TFF3 solution at a molar ratio of 3:1 (Cy5.5:TFF3) and incubated for 30 min at 37 °C in a thermostat protected from light. Free dye was removed using 3KD ultrafiltration tubes. Cy5.5-TFF3@Rqdl10 (Rqdl10 10 mg/mL) was prepared as previously described. Next, 50 µL of Cy5.5-TFF3@Rqdl10 was placed in a 1.5 mL EP tube, and 200 µL of PBS was then added. At the indicated time points (0 h, 12 h, 24 h, 36 h, 48 h), the supernatant was removed and Cy5.5-TFF3@Rqdl10 in the EP tube was visualized using an in vivo imaging system (VILBER, Collégien, France).

### 4.7. In Vivo Imaging of Cy5.5−TFF3, FITC−Rqdl10 Hydrogel, and Cy5.5−TFF3@Rqdl10 in Mice

BALB/c nude mice were fasted for 24 h and abstained from drinking for 12 h. They were then anesthetized with sodium pentobarbital (intraperitoneally, 50 mg/kg), and a midline epigastric incision was made to fully expose the gastric tissue. Sequentially, 100 µL of Cy5.5-TFF3, FITC-Rqdl10 hydrogel (10 mg/mL), and Cy5.5-TFF3@Rqdl10 were injected into the gastric lumen to adhere to the gastric mucosa as much as possible, after which the incision was sutured. The nude mice were anesthetized at the indicated time points (0 h, 12 h, 24 h, and 36 h) after the injections, and the measurements were performed using the in vivo imaging system (VILBER, France).

### 4.8. Cell Culture and Treatment

Human gastric mucosal epithelial cells (GES-1 cells) were procured from Saibekang Biotechnology (Shanghai, China). GES-1 cells were cultured routinely in DMEM supplemented with 10% FBS and 1% penicillin and streptomycin, maintaining them in a humidified incubator at 37 °C with 5% CO_2_. The cytocompatibility of the Rqdl10 hydrogel was assessed using the CCK8 method. A 100 μL cell suspension was inoculated into a 96-well plate, followed by the addition of 25 μL of Rqdl10 solution and 25 μL of PBS. The mixture was gently mixed to ensure encapsulation of GES-1 cells into the Rqdl10 hydrogel. The proliferation and toxicity test were conducted at cell densities of 5 × 10^3^ cells/well and 2 × 10^4^ cells/well. After 1, 2, and 3 days, 10 μL of CCK8 was added and incubated for 2 h, followed by absorbance measurement at 450 nm to calculate cell viability. GES-1 cells were seeded in 96-well plates at a density of 5 × 10^3^ cells/well and pretreated with TFF3, Rqdl10 hydrogel, or TFF3@Rqdl10 for 24 h. Subsequently, acute injury was induced in GES-1 cells by co-incubation with 6% ethanol DMEM medium for 4 h. Changes in inflammation and apoptosis−related proteins in GES-1 were analyzed using Western Blotting.

### 4.9. Cell Scratch Assay

Assessment of potential cell migration capacity was conducted using the cell scratch method. In the absence of ethanol stimulation, GES-1 cells were cultured with TFF3, Rqdl10 hydrogel, or TFF3@Rqdl10 for 24 h, and cell scratches were created on the cell layer using a cell scraper. The cell debris was washed with PBS, and photographs were captured at 0 h, 12 h, 24 h, 36 h, and 48 h to measure the size of the scratches. Under ethanol exposure, GES-1 cells were treated with TFF3, Rqdl10 hydrogel, or TFF3@Rqdl10 for 24 h, followed by treatment with 6% ethanol DMEM medium for 4 h. Subsequently, cell migration was observed under a microscope at 0 h, 12 h, 24 h, 36 h, and 48 h, and the scratch area was assessed using ImageJ.

### 4.10. Evaluation of Anti-Apoptosis Effects In Vitro

Detection of apoptosis levels in GES-1 cells was performed using the Calcein-AM/PI Apoptosis Detection Kit (Beyotime, China). Briefly, GES-1 cells were seeded into 96-well plates at a density of 5 × 10^3^ cells per well. After treatment with various experimental conditions, the cells were washed once with PBS, and then 100 μL of the prepared Calcein-AM/PI fluorescent staining solution was added to each well. The plates were then incubated at 37 °C for 30 min in the dark. Subsequently, the cells were examined under a fluorescence microscope. For flow cytometry analysis, GES-1 cells were seeded into 6-well plates at a density of 1 × 10^6^ cells per well. After treatment with different experimental conditions, the cells were collected into centrifuge tubes according to their respective groups. A 1 mL cell suspension (1 × 10^6^ cells/mL) was centrifuged at 1000 rpm for 5 min, and then 1 mL of Calcein-AM/PI assay working solution was added. After resuspending the cells as a single-cell suspension, they were incubated at 37 °C in the dark for 30 min. Finally, the cells were analyzed using a flow cytometer (CytoFLEX, Beckman, Brea, CA, USA).

### 4.11. Acetic Acid-Induced Gastric Injury in Rats

Adult healthy SD rats weighing 200–250 g were selected and included in the experiment. The animals were housed under standardized conditions, including controlled temperature, humidity, and a 12 h light–dark cycle, and were provided with standard feed and tap water ad libitum. All procedures related to animal experiments were approved by the Experimental Animal Ethics Committee of Chongqing Medical University. Acetic acid (AcOH) was used to induce gastric mucosal injury in rats, following the method reported by Kang [55]. Briefly, SD rats were randomly divided into the control group, AcOH group, AcOH + TFF3 group, AcOH + Rqdl10 group, and AcOH + TFF3@Rqdl10 group. The rats were fasted for 24 h before surgery, and then anesthetized with sodium pentobarbital (50 mg/kg) by intraperitoneal injection to fully expose the gastric tissue. A circular EP tube with a diameter of approximately 10 mm was positioned adjacent to the serosa of the gastric antrum. Subsequently, 0.1 mL of 100% glacial acetic acid was injected into the tube, where it was allowed to remain for 60 s. Following this, the acetic acid was aspirated, and the serosa was rinsed twice with PBS. Each of the anterior and posterior walls of the gastric antrum underwent modeling once, resulting in the formation of two ulcers with symmetrical size and location. The posterior wall served as the AcOH group, while the anterior wall was designated as the experimental group. In the AcOH group, 100 μL of PBS was injected into the ulcer, whereas in the AcOH+TFF3 group, 100 μL of TFF3 solution was injected into the ulcer. Similarly, the AcOH + Rqdl10 group received an injection of 100 μL of Rqdl10 hydrogel into the ulcer, and the AcOH + TFF3@Rqdl10 group received 100 μL of TFF3@Rqdl10 into the ulcer. Following the procedure, the omentum was covered, and the peritoneal, abdominal wall tissues, and skin were intermittently sutured. The rats were then housed individually. After a period of 5 days, the rats were sacrificed under anesthesia.

### 4.12. Western Blotting

Proteins were extracted from gastric tissues or GES-1 cells using radioimmunoprecipitation assay (RIPA) lysis buffer (Beyotime, Shanghai, China), and protein quantification was conducted using the BCA method (Beyotime, China). Equal amounts of proteins were then separated on a 10% SDS-PAGE gel and subsequently transferred to a polyvinylidene difluoride (PVDF) membrane. Following blocking for 10 min at room temperature using rapid blocking solution (Ncmbio, Suzhou, China), PVDF membranes were incubated with primary antibodies overnight at 4 °C. Afterward, the PVDF membrane was washed three times using tris−buffered saline (TBST) and incubated with secondary antibody for 1 h at room temperature. Finally, emitter-coupled logic (ECL) substrate (Ncmbio, China) was added dropwise, and the membrane was observed using a chemiluminescent detection system (Bio-Rad, Hercules, CA, USA). Quantification of protein bands was performed using Image J software (1.53K). The antibodies used were as follows: β-actin (AB0035, 1:5000, Abways, Beijing, China), Bax (A20227, 1:2000, ABclonal, Wuhan, China), Bcl2 (A20777, 1:1000, ABclonal, China), TNF−α (ER1919-22, 1:500, HuaBio, Hangzhou, China), IL−6 (R1412-2, 1:500, HuaBio, China), and NF−κB (PSH0−27, 1:1000, HuaBio, China).

### 4.13. Statistical Analysis

All data were presented as mean ± SD (*n* = 3) and analyzed using a one-way ANOVA test followed by the Tukey honest significant difference (HSD) test using SPSS software (version 22, Chicago, IL, USA), and *p* < 0.05 was considered a significant difference.

## 5. Conclusions

In this study, we engineered an optimal drug delivery vehicle, Rqdl10 hydrogel, for TFF3. The Rqdl10 hydrogel exhibits excellent biocompatibility, enabling the sustainable and slow release of TFF3. Moreover, the Rqdl10 hydrogel effectively protects the gastric mucosa through a physical barrier action similar to that of gastric mucus, mitigating damage caused by ethanol or gastric acid, thereby providing an optimal healing microenvironment for gastric mucosal repair. Compared to the treatment with TFF3 or Rqdl10 hydrogel alone, the TFF3@Rqdl10 system combines the physical barrier function of the Rqdl10 hydrogel and the biological activity advantages of TFF3, promoting the repair of gastric mucosal damage through multi−level and multifaceted actions, including the promotion of the proliferation and migration of gastric mucosal epithelial cells, and the inhibition of inflammation and apoptotic responses. These findings underscore the promising therapeutic potential of self−assembled peptide Rqdl10 hydrogels in biomedical applications, potentially revolutionizing healthcare science. 

## Data Availability

The data presented in this study are available in the article.
